# A man with recurrent fever, arthritis, and rashes-brucellosis? A case report

**DOI:** 10.1186/s12879-019-4746-0

**Published:** 2020-01-07

**Authors:** Wen Wang, Xu Lu, Chengbo Li, Myong Jun Ri, Wei Cui

**Affiliations:** 1 Department of Infectious Diseases, The First Affiliated Hospital of China Medical University, 155 Nanjing North Street, Shenyang, 110001 Liaoning Province China; 2 Department of Infectious Diseases, Clinical Faculty Number 1, Kim Il Song University Pyongyang Medical College, Pyongyang, Democratic People’s Republic of Korea

**Keywords:** Brucellosis, Myelodysplastic syndrome, Neutrophilic dermatosis, Sweet’s syndrome

## Abstract

**Background:**

We report a rare case of chronic brucellosis accompanied with myelodysplastic syndrome and neutrophilic dermatosis, which to the best of our knowledge, has never been reported.

**Case presentation:**

A young man was admitted to our hospital complaining of recurrent fever, arthritis, rashes and anemia. He had been diagnosed with brucellosis 6 years prior and treated with multiple courses of antibiotics. He was diagnosed with myelodysplastic syndrome and neutrophilic dermatosis following bone marrow puncture and skin biopsy. After anti-brucellosis treatment and glucocorticoid therapy, the symptoms improved.

**Conclusions:**

Clinicians should consider noninfectious diseases when a patient who has been diagnosed with an infectious disease exhibits changing symptoms.

## Background

Brucellosis infections usually exhibit polymorphic features affecting all organ systems. Although hematological involvement and rashes can occur during brucellosis, serious clinical disease is rare. Herein, we report a case of recurrent fever, arthritis, rashes and severe anemia. Besides the patient’s previous diagnosis of brucellosis, we attributed his new conditions to myelodysplastic syndrome and neutrophilic dermatosis.

## Case presentation

A 32-year-old male was admitted to our hospital on July 13, 2018. The patient’s parents were second cousins. His physical and mental development was normal and he frequently consumed kebabs. The patient began to experience repeated fevers in April, 2013. The temperature was typically 38.5 °C in the afternoon and at night, and was accompanied by mandibular pain. The patient underwent osteomyelitis curettage in a local hospital and his brucellosis antibody test was positive (the titer was unknown). The patient was then treated with levofloxacin, cefodizime, and oxytetracycline for three courses (half a month on each course and half a month between courses). He gradually felt pain in the right shoulder joint, left elbow joint, right hip joint, and left knee joint, for which he frequently took analgesic tablets to relieve the pain. In January 2014, the patient was admitted to another hospital due to fever and joint pain. His brucellosis antibody was 1:100 positive, his C-reactive protein (CRP) was 104 mg/L (0–8), and blood routine was normal. A bone marrow puncture showed bone marrow proliferating actively. Positron-emission tomography-computed tomography (PET-CT) showed bone destruction in the mandible, bilateral humerus, right greater trochanter of femur, and increased fluorine-18-fluorodeoxyglucose (FDG) metabolism (SUVmax = 10.06). The results of PET-CT and clinical examinations indicated that the patient suffered from an infectious disease. The diagnosis was “brucellosis” and the fever was relieved after 3 months of treatment with moxifloxacin + rifampicin + doxycycline. However, joint pain persisted and the brucellosis antibody test remained positive.

In 2015, a left upper limb bone scraping biopsy was performed and diagnosed as “Fibrous dysplasia of bone”. In 2016 and 2017, the patient visited several hospitals to treat the brucellosis; however, his osteoarticular symptoms continued to worsen.

**Fig. 1:**
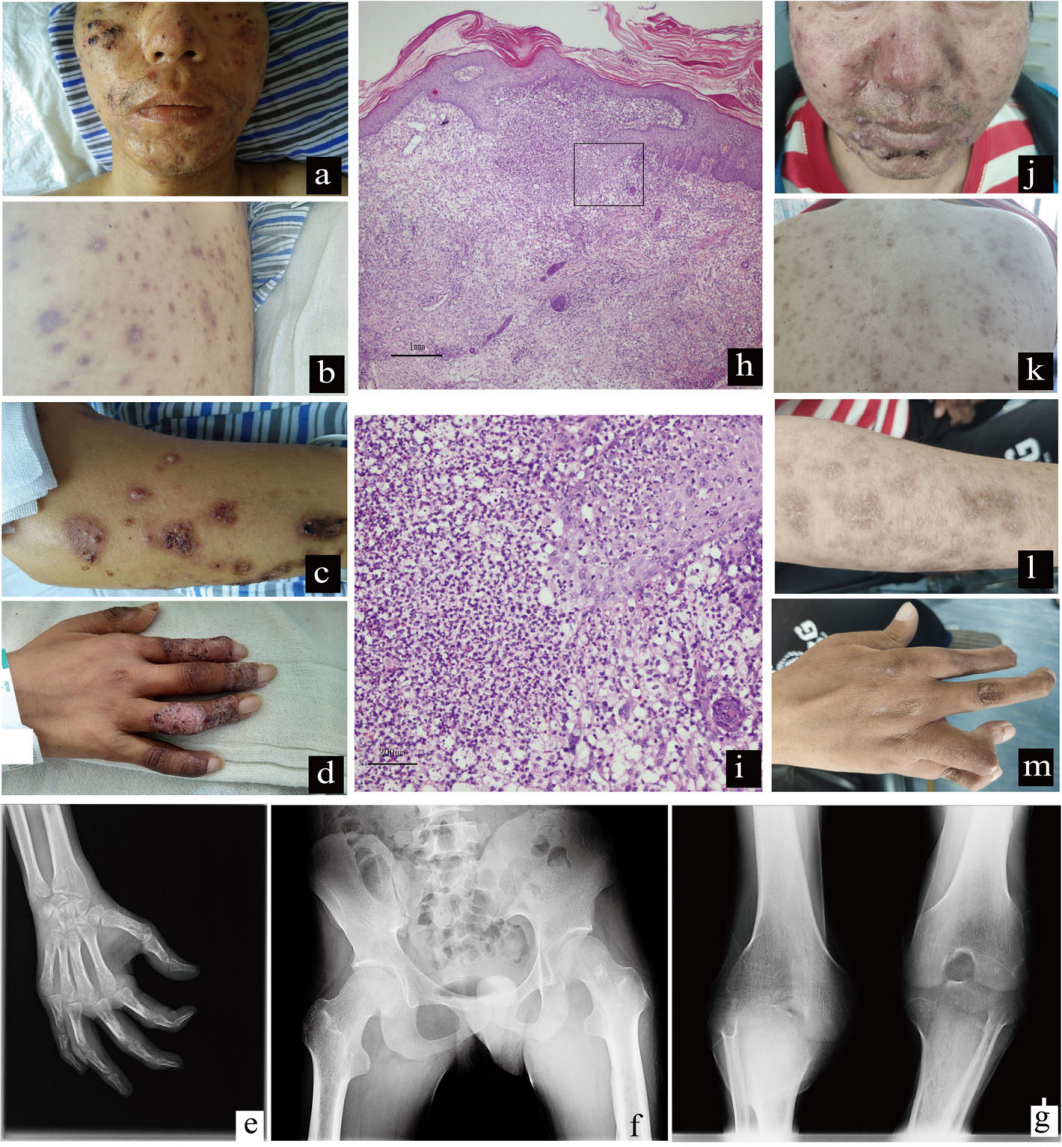
Rashes, skin biopsy and X-ray films of the patient’s joints. **a**-**d** Rashes before treatment. There were intensive brown-red papules and a partial scar on the face, trunk and limbs. Some scabs and scars, pustules at the tip of the finger. **e** X-ray of the right hand. The bone density of all bones was reduced unevenly, the interphalangeal space became narrow, and the soft tissue was slightly swollen. **f** X-ray of the bilateral hip joint. The cortex of the left upper femur was slightly thickened, and the density of the medullary cavity was not uniform. **g** X-ray of the bilateral knee joint. The joint space was narrowed. **h**-**i** Skin biopsy of the right upper limb. H&E staining (**h**: 40× magnification, **i**: 200× magnification): diffuse and dense infiltration in the dermis, which was mainly neutrophil infiltration and nuclear fragmentation, irregular proliferation of the upper epidermis, and a superficial scab. **j**-**m** Rashes after glucocorticoid treatment for 10 months. Acne occurred on the face and back. Rashes on the extremities were resolved and pigmentation subsided

Since 2018, swelling gradually developed, and the patient experienced pain and deformities in his hands and knees, and was unable to walk. Facial rashes appeared with itchiness, but the patient did not use antibiotics. In July 2018, the rashes spread throughout the body (Fig. 1a-d), the fever was aggravated, blood hemoglobin (Hb) decreased to 36 g/L, and platelet (PLT) count decreased. Following blood transfusion, the patient attended our hospital.

Following admission in our hospital, the patient’s blood cultures were negative and his standard tube agglutination test (SAT) was 1:400 positive. The blood routine showed that white blood cell (WBC) count was normal, Hb was 43–63 g/L (130–175), and the PLT count was 58–104*10^9 /L (125–350). CRP was 130–200 mg/L (0–5). Blood biochemical examinations revealed that alkaline phosphatase levels were slightly elevated. The T cell enzyme-linked immono-spot assay for tuberculosis was negative. His autoantibody series (including ANA, anti-dsDNA, anti-SM, anti-U1RNP, anti-SSA, anti-SSB, anti-SCL 70, anti JO-1, RO-52, PM-Sd, AMA-M2, AHA, ANUA, anti-ribosomal P protein antibody), anti-cyclic citrullinated peptide antibody (CCP), and antineutrophil cytoplasmic antibodies (ANCA) were also negative. X-ray of the right hand (Fig. 1e) showed that the bone density of all bones was reduced unevenly, the interphalangeal space became narrow, and the soft tissue was slightly swollen. X-ray of the bilateral hip joint (Fig. 1f) showed that the cortex of the left upper femur was slightly thickened, and the density of the medullary cavity was not uniform. X-ray of the bilateral knee joint (Fig. 1g) showed that the joint space was narrowed. Bone ECT showed that the metabolism of the long bones and joints of the four limbs were increased, as was the metabolism of the hands, maxilla and mandible. Chest and abdominal imaging examinations were approximately normal.

To clarify the cause of fever, rashes and severe anemia, we performed bone marrow and skin biopsies. Skin biopsy showed diffuse and dense infiltration in the dermis, which was mainly neutrophil infiltration and nuclear fragmentation, irregular proliferation of the upper epidermis and a superficial scab (Fig. 1h-i). The pathological diagnosis was neutrophilic dermatosis. A bone marrow smear showed that the proliferation of the granulocyte system was active, while the proliferation of the erythrocyte system decreased, mainly in late and middle-aged erythrocytes. Occasional, large, multinucleated middle and late erythrocytes were observed, nucleus sprouting and nuclear fragmentation was observed in late erythroblasts, and the sizes of mature red blood cells varied. The ratio of lymphocytes decreased and cell morphology was normal. The patient was diagnosed with myelodysplastic syndrome with single lineage dysplasia (MDS-SLD). In the following genetic examinations, a p.g642 fs insertion mutation in the *ASXL1* gene was detected, and his chromosomal examination showed 46,XY,der(12)t(1;12)(q11;p11)[15]/46,XY[5].

The patient was given anti-brucellosis therapy (ceftriaxone + levofloxacin + rifampicin + doxycycline) for 6 months and glucocorticoid therapy. The dosage of methylprednisolone was 60 mg once per day, which was then gradually decreased according to the symptoms. By the end of May 2019, the glucocorticoid had been used for 10 months. The current dose of methylprednisolone is 8 mg per day by oral administration. At present, the patient has no fever, most rashes have been resolved and pigmentation has subsided (Fig. 1j-m). The patient’s joint pain in his hands and knees has been relieved; however, the hands and knees remain deformed. The patient also remains unable to stand. The Hb increased to 121 g/L, while the PLT count remained low, at 49*10^9 /L (125–350).

## Discussion and conclusions

Brucellosis is caused by bacteria of the genus, *Brucella*, which are major zoonotic pathogens, worldwide. Human brucellosis is a major public health concern that has re-emerged in China since the mid-1990s. The highest recorded number of cases (56,989 cases) was in 2015, and brucellosis infections usually have polymorphic features affecting all organ systems.

In a retrospective analysis of 1028 brucellosis cases in Turkey [1], 13.6% of cases were evaluated as chronic (> 12 months). In a retrospective study of 2041 brucellosis patients in China [2], 4% of patients were chronic. The most common clinical manifestations of chronic brucellosis are arthralgia, fatigue, back pain, sweating, fever and limited motion. In the same Chinese study of 2041 brucellosis patients, there was evidence of osteoarticular involvement in 87% of chronic brucellosis patients [2], including peripheral arthritis (75%), spondylitis (33%) and sacroiliitis (2%). In our report, the young man frequently consumed kebabs and his family raised sheep. He experienced recurrent fever and arthritis since 2013, and his SAT was 1:400 positive. Based on these findings, the patient was diagnosed with chronic brucellosis. We inferred that the recurrence of brucellosis in the patient may have been related to incomplete prior treatments.

Although hematological involvement is frequent in brucellosis, most of such cases are mild, and serious clinical disease is rare. The most commonly reported abnormal laboratory tests are aleucocytosis, anemia, thrombocytopenia, pancytopenia and leukocytosis [3]. The patient had severe anemia and his Hb was 36 g/L, which was difficult to explain with brucellosis. Thus, we performed bone marrow aspiration to confirm the diagnosis of MDS. We attributed his severe anemia to multiple factors, including brucellosis, MDS, and malnutrition related to repeated fever. Brucellosis, in association with hematological malignancies, such as acute myeloid leukemia and acute lymphoblastic leukemia, has been reported [4]. Although rare, acute brucellosis associated with MDS has been reported [5]. Moreover, the relationship between the treatment for brucellosis and MDS remains unknown. There is a case report of secondary MDS caused by cyclophosphamide or rifampicin [6]; however, in this case, there was no significant time correlation between MDS and the treatment with drugs. The patient also contained a p.g642 fs insertion mutation in the *ASXL1* gene, combined with chromosomal abnormalities; thus, we considered that MDS was primary MDS instead of secondary to brucellosis or drugs.

Rashes can occur during brucellosis, and are primarily observed in acute cases. The frequency of skin manifestations varies widely in studies, ranging between 3.8 and 17 %[7]. Four main clinical patterns of skin lesions associated with brucellosis have been described, including disseminated papulonodular eruption, diffuse maculopapular rash, and erythema nodosum-like and purpuric lesions. Dermal perivascular and periadnexal infiltrates of lymphocytes and histiocytes with a focally granulomatous appearance, with or without multinucleated giant cells, have been reported as characteristic histopathologic findings of skin lesions of *B. melitensis* infections [8]. To identify the cause of the rashes, we performed a skin biopsy, which confirmed the diagnosis of neutrophilic dermatosis.

Neutrophilic dermatoses are a group of conditions characterized by the accumulation of neutrophils in the skin and clinically presenting with polymorphic cutaneous lesions, including pustules, bullae, abscesses, papules, nodules, plaques and ulcers. Neutrophilic diseases may be subdivided into three main groups [9]: pyoderma gangrenosum (PG), Sweet’s syndrome (SS), and amicrobial pustulosis of the folds. This patient was considered as having SS. The patient had acute onset, high fever, and there were intensive brown-red papules and a partial scar on the face, trunk and limbs. Some scabs and scars, pustules at the tip of finger, and a dense neutrophilic infiltrate in the dermis were also observed. SS can be classified into classic, malignancy-associated, and drug-induced SS, depending on the clinical setting in which the disease develops. Approximately 85% of reported cases of malignancy-associated SS have underlying hemopoietic neoplasia, such as acute myeloblastic leukemia, myeloproliferative neoplasms, MDS, and myelofibrosis [10]. In our case, we inferred SS as a paraneoplastic manifestation of MDS. The absence of neutrophilia in this patient is possible because of the association between SS secondary to MDS.

In terms of treatment, although the patient’s blood culture was negative, we decided to administer anti-brucellosis treatment for 6 months given the low positive rate of blood culture for chronic brucellosis [2], and that his SAT titer was 1:400 positive. For SS, the first-line treatment is glucocorticoid. After glucocorticoid treatment, the patient’s body temperature gradually returned to normal, the rashes improved significantly, and joint swelling and pain was alleviated; however, joint deformity remained obvious. Thus, we hypothesize that relief of the patient’s symptoms may be attributed to the combination of the anti-brucellosis treatment and glucocorticoid. The patient remains in the process of follow-up.

One limitation of our case is that we did not identify any etiological evidence for brucellosis, and we had no PCR detection conditions for *Brucella*. Another limitation is that we cannot confirm whether the genetic mutation was primary or secondary to long-term infection with *Brucella*.

In conclusion, we report a case of recurrent fever, arthritis, rashes and anemia, which was diagnosed as chronic brucellosis, MDS, and SS. Our case is rare, but may inform clinicians to consider noninfectious diseases when a patient exhibits unexplainable conditions and has had a previous infectious disease.

## Data Availability

Not applicable (no datasets were generated or analyzed during the current study).

## Abbreviations

ANCA: Antineutrophil cytoplasmic antibodies
CCP: Cyclic citrullinated peptide
CRP: C-reactive protein
FDG: Fluorodeoxyglucose
Hb: Hemoglobin
MDS-SLD: Myelodysplastic syndrome with single lineage dysplasia
PET-CT: Positron-emission tomography-computed tomography
PG: Pyoderma gangrenosum
PLT: Platelet
SAT: Standard tube agglutination test
SS: Sweet’s syndrome
WBC: White blood cell

## References

[1] Buzgan T, Karahocagil MK, Irmak H, Baran AI, Karsen H, Evirgen O (2010). Clinical manifestations and complications in 1028 cases of brucellosis: a retrospective evaluation and review of the literature. Int J Infect Dis.

[2] Shi Y, Gao H, Pappas G, Chen Q, Li M, Xu J (2018). Clinical features of 2041 human brucellosis cases in China. PLoS One.

[3] Zheng R, Xie S, Lu X, Sun L, Zhou Y, Zhang Y (2018). A systematic review and meta-analysis of epidemiology and clinical manifestations of human brucellosis in China. Biomed Res Int.

[4] Sari I, Altuntas F, Hacioglu S, Kocyigit I, Sevinc A, Sacar S (2008). A multicenter retrospective study defining the clinical and hematological manifestations of brucellosis and pancytopenia in a large series: hematological malignancies, the unusual cause of pancytopenia in patients with brucellosis. Am J Hematol.

[5] Li JJ, Sheng ZK, Tu S, Bi S, Shen XM, Sheng JF (2012). Acute brucellosis with myelodysplastic syndrome presenting as pancytopenia and fever of unknown origin. Med Princ Pract.

[6] Tsuboi E, Narui K, Nakatani T, Nakamori Y, Nakata K, Muto Y (1992). A case of Legionella pneumonia with myelodysplastic syndrome. Nihon Kyobu Shikkan Gakkai Zasshi.

[7] Ayaslioglu E, Kocxak M, Bozdogan O (2009). A case of brucellosis presenting with widespread maculopapular rash. Am J Dermatopathol.

[8] Ariza J, Servitje O, Pallarés R, Fernández Viladrich P, Rufí G, Peyrí J (1989). Characteristic cutaneous lesions in patients with brucellosis. Arch Dermatol.

[9] Marzano AV, Borghi A, Wallach D, Cugno M (2018). A comprehensive review of neutrophilic diseases. Clin Rev Allergy Immunol.

[10] Raza S, Kirkland RS, Patel AA, Shortridge JR, Freter C (2013). Insight into Sweet's syndrome and associated-malignancy: a review of the current literature. Int J Oncol.

